# Healthcare career pathways: a case study of strengthening health workforce and empowering marginalised women in Bihar, India

**DOI:** 10.3389/frhs.2026.1695441

**Published:** 2026-02-23

**Authors:** J. Narang, C. Ratcliff, V. Kanth

**Affiliations:** 1 Consultant, Edinburgh, United Kingdom; 2 EMMS International, Edinburgh, United Kingdom; 3 EMMS International to Develop HCP, Bihar, India

**Keywords:** Bihar, gender, health education, health workforce, India, investment in health, workforce development models

## Abstract

The Healthcare Career Pathways (HCCP) programme, implemented by EMMS International and Duncan Hospital in Bihar, India, addresses dual public health challenges: the shortage of long-term healthcare workforce in hard-to-staff health facilities, and the systemic exclusion of vulnerable women from higher education and professional employment. Since its expansion in 2019 to Bihar, India, HCCP has ensured targeted advocacy and selection, mentoring, financial support to pursue accredited healthcare qualifications, and guaranteed employment to economically and socially vulnerable women. This case study presents the HCCP model as implemented in Raxaul, Bihar, India, including its outcomes between 2019 and 2025, impacts, strengths and challenges. It is based on the findings of a comprehensive, independent, objective evaluation of HCCP, which was conducted using quantitative review of programme records and documents, qualitative study design comprising semi-structured interviews and focus groups, and thematic analysis of the data. By the year 2025, HCCP has supported 54 economically and socially marginalised young women in healthcare training and qualifications, with 24 of these graduates employed at The Duncan Hospital. The evaluation findings reveal substantial impacts on the young women and their families' economic independence and self-efficacy; gender norms including delayed marriage and reduction in dowry demands; and shifts in community perceptions and attitudes towards girls' marriageability, education and employment particularly in the healthcare sector as well as intergenerational educational and childbirth aspirations whereby families now welcome the birth of a girl child rather than opposing it. Key challenges include limited geographical reach, partial financial support, and narrowing of career aspirations to nursing among the young women and families. This evaluation demonstrates HCCP as an evidence-based model with potential for replicability and sustainability in lower and middle-income countries to strengthen health systems in rural and underserved regions through workforce development and guaranteed recruitment in the local geographical setting while advancing gender equity and empowerment aligned with the sustainable development goals. As of 2025, HCCP is already being replicated in Madhipura Hospital in Bihar and Jiwan Jyoti Hospital in Uttar Pradesh in India. Recommendations focus on positioning HCCP within the local socio-cultural and geographical context, diversifying career pathways, strengthening mentorship, and appropriate follow-up, monitoring and evaluation mechanisms.

## Introduction

The shortage of qualified healthcare workforce globally presents a persistent and critical barrier to achieving universal health coverage (1). The World Health Organisation (WHO) projects a shortage of 11 million health workers by 2030, mostly in low- and lower-middle-income countries (LMICs) (2). It also emphasises the global challenge of deploying and retaining health workers in rural and remote areas that affects access to equitable health services. Moreover, persistent gender inequalities, limited women's empowerment, systemic undervaluation of health and care work predominantly performed by women, and lack of investment in the health workforce development for education and training have been emphasised as barriers to creating sustainable employment opportunities, particularly for women and youth; and investment in these areas is considered critical for achievement of the sustainable development goals (SGDs) (2, 3). These challenges are evident in India, where healthcare workforce shortages, including doctors and nurses, are acute especially in poor, remote and underserved regions, and recruitment and retention of competent and long-term staff remains a systemic challenge - with persistent disparities in female economic participation, women's health outcomes and women's agency (4). India falls well below the threshold set by the WHO of 44.5 doctor, nurses and midwives per 10,000 population (5). India has a very low density of health workers per 10,000 population, with stock density of doctor and nurses/midwives being 8.8 and 17.7 respectively per 10,000 persons and active health workers' density is estimated to be even lower at 6.1 and 10.6 respectively; and when accounting for adequate qualifications, the estimates drop further down to 5.0 and 6.0 respectively, along with low levels of skill-mix that is doctor-centric with a lower number of nurses (6). Further, the distribution of the health workforce across the Indian states is highly skewed, with some of the lowest estimates in the less developed states such as Bihar where acute shortage of health workforce is reported. Bihar is the third most populous state in India, where the health expenditure per capita is approximately one-third of the national average and the density of health workers, primarily doctors and nurses, is estimated to be 3.3 and 2.0 respectively per 10,000 population compared with the recommended 7 (4, 6–8). Compared with the all India nurse-to-doctor ratio of 1.7:1, Bihar is reported to have less than 1 nurse (0.4) per doctor in Bihar, which is the lowest in the country (6). Substantial inter- and intra-state disparities in health outcomes are observed in Bihar relative to the rest of India, shaped by limited access to healthcare and education and intersecting with gender-based disparities. Bihar is reported to have some of the worst gender-related social indicators in the country, frequently ranking among the lowest in India. For example, the sex ratio at birth in Bihar is 908 girls to every 1000 boys compared with 929 across the whole of India (9), female literacy rates are recorded as 60.5% compared to male literacy rates of 79.7%, and Bihar's female workforce participation rate is estimated as 2.7% compared with the nationwide male participation of 73%–74% (4, 10). These systemic disparities not only impede female economic participation, women's empowerment and agency, but also limit health workforce capacity (4, 6).

The Healthcare Career Pathways (HCCP) programme is implemented by EMMS International in partnership with The Duncan Hospital in Raxaul, East Champaran District of Bihar, India since August 2019. By offering vulnerable young women pathways into accredited healthcare education along with guaranteed employment at The Duncan Hospital, HCCP addresses the twin challenges of the shortage of qualified healthcare workers, particularly nurses, in poor, remote and underserved regions including difficulties with recruitment and retention as well as gender-based disparities in access to higher education and professional employment. An independent, objective evaluation of the HCCP's implementation at The Duncan Hospital was undertaken with the key objectives of understanding the model, its implementation within the local geographical context, key outcomes, impacts, efficacy, challenges, and replicability. Based on the findings of the evaluation, this case study presents the HCCP as a viable model within the context of rural health system strengthening and gender equity. Prior research has documented similar interventions, such as community-based training schemes targeting female community health workers (11), which demonstrate improvements in retention and local service delivery. Furthermore, studies exploring mentorship and local bond arrangements in healthcare training (12) echo components of HCCP's design and affirm their potential effectiveness in fostering sustainability. Aligned with the WHO's strategy of widely sharing health workforce development models to achieve the SDGs (2), this case study seeks to contribute to the emerging body of literature, policy and practice by providing an in-depth analysis of the HCCP's implementation including its background and contextual framework as well as findings of the evaluation undertaken to highlight the outcomes, impact, limitations, replicability, sustainability and recommendations.

As of 2025, HCCP is already being replicated in two other hospitals in India by EMMS –Jiwan Jyoti Hospital and Madhipura Hospital. This demonstrates HCCP as an evidence-based model that has a wide scope of replication and sustainability.

## Key objectives and methodology of the evaluation

### Key objectives

Several concerns and questions are central to the success of the initiative that guided the evaluation and present case study. With this context, the key objectives of the evaluation were to:

Examine the extent to which the HCCP enhances access to professional healthcare training for women from the most marginalised socio-economic backgrounds;
Investigate the effectiveness of the programme in retaining trained graduates within a local hard-to-staff healthcare facility;
Explore the social, economic, and gender-related impacts of the programme on the participants, their families, and their communities;
Understand the significant components of the programme model that are most critical to its sustainability and replicability.

### Methodology

#### Design, participants, and setting

**Figure 1:**
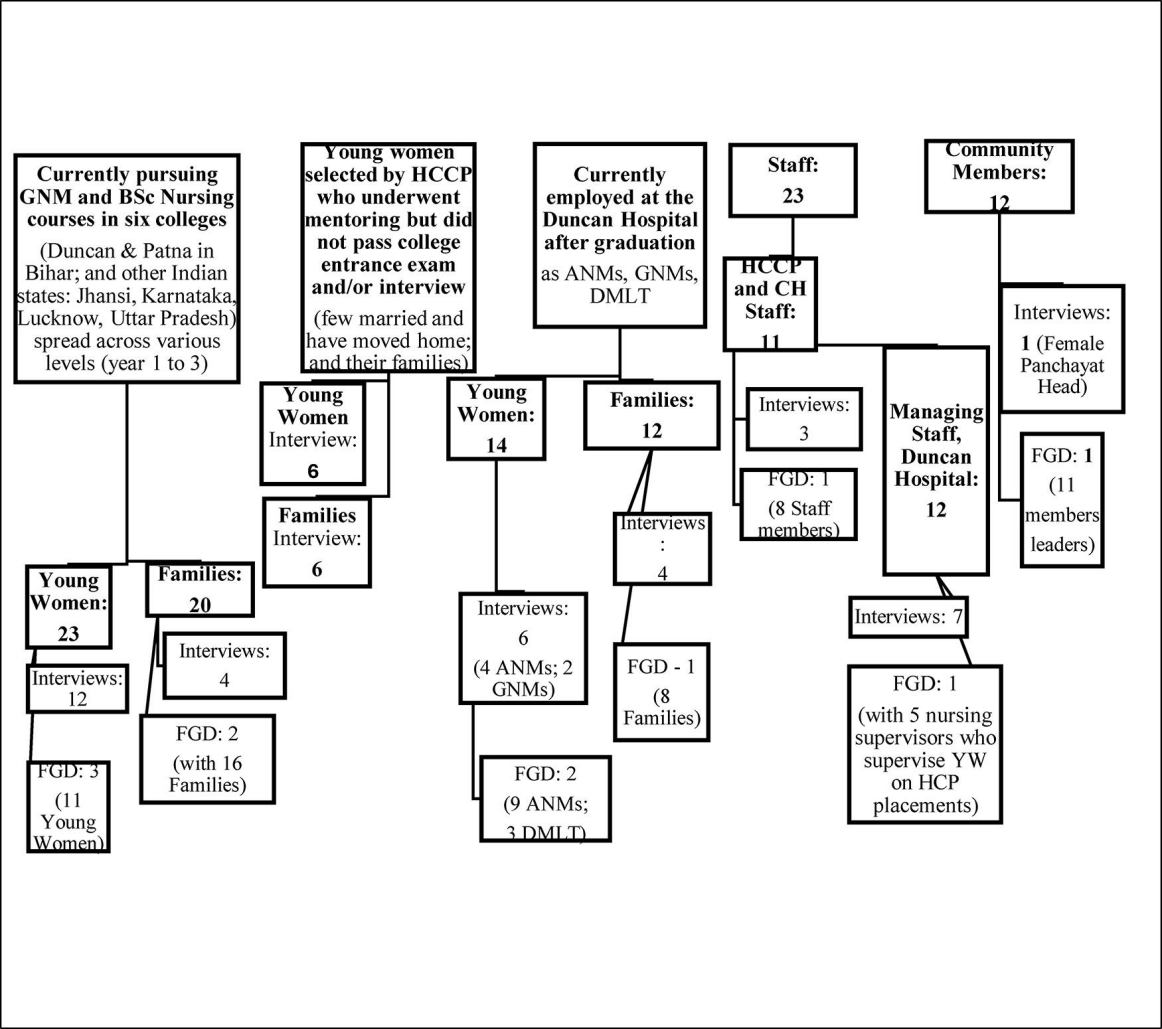
Broad overview of the participants: categories and numbers covered.

The HCCP programme evaluation was conducted primarily following a qualitative study design that included 54 in-depth, semi structured interviews and 10 focus group discussions (FGDs). Participants included various categories of young women and their families based on their involvement with the HCCP, staff, and community members (Figure 1 provides an overview of various categories and numbers covered), who were invited by the HCCP and The Duncan hospital staff to participate voluntarily in the evaluation. Face-to-face interviews and FGDs were conducted at locations comfortable for participants including their homes and/or workplace (The Duncan Hospital) with their verbal and written consent. In addition, 6 interviews (out of 54) and 1 FGD (out of 10) were conducted online with young women studying in institutions in other states of India, outside Bihar. The interviewer was the same throughout, who is also from India, who ensured sensitivity, consistency and continuity, both face-to-face and online. Each interview and FGD lasted between 60 and 90 min and captured participants' views, experiences, feelings and reactions regarding the HCCP process and their engagement with it; benefits, impacts; challenges; gaps; and recommendations. Emerging themes were confirmed and explored further through subsequent interviews and FGDs. Quantitative data was also gathered, collated and synthesised through programme documents and records from August 2019 - December 2025 that provided a deeper understanding of the HCCP model's process, implementation, and the number of young women and families covered and affected.

#### Data analysis

Thematic analysis of the data was undertaken following the Braun and Clark (13) approach for identifying patterns across data, which included familiarisation with the data, generating initial codes systematically, grouping codes into potential themes, reviewing and refining themes, defining, naming and finalising themes, leading to writing the final report supported by quotations from the data. Data were compared and analysed iteratively until the emerging themes adequately captured the content and the final themes closely reflected the data, that is, until thematic saturation was reached (13, 14).

## The HCCP model

### Background and rationale

EMMS International (EMMS) is a Scottish charity that works in partnership to strengthen healthcare in India, Malawi, Nepal, Rwanda, Scotland and Zambia. EMMS and its partners focus on strengthening healthcare systems in management of non-communicable diseases (especially developing holistic palliative care) and working to increase the global healthcare workforce (15). The Duncan Hospital, based in Raxaul, Bihar, India is a unit of the Emmanuel Hospital Association (EHA), a not-for-profit registered organisation that provides specialised healthcare in some of the most remote and underdeveloped areas of India and is committed to the transformation of communities with programmes focussing on health and wellbeing, development and empowerment of women (16, 17).

Implemented by EMMS in partnership with The Duncan Hospital, HCCP's strategic aims are twofold:

To ensure a sustainable, locally sourced supply of trained healthcare workforce committed to long-term service in hard-to-staff health facilities; and
To support women of vulnerable background into and through healthcare college or university and into guaranteed jobs.

EMMS has implemented HCCP in India, Malawi, Nepal and Zambia, although HCCP models differ between countries, depending on funding and country context. EMMS launched HCCP in India in August 2019 in The Duncan Hospital for young women in the area, recognising the hospital's challenges of attracting and retaining staff in the poor town of Raxaul, with its geographic remoteness, limited career progression opportunities, and socio-cultural disincentives. Parallel to this is the entrenched gender gap in professional healthcare roles and overall socio-cultural gender disparities underpinned by: early marriage; dowry demands; financial dependence; restricted mobility; limited access to quality secondary, tertiary and specialist professional education; lack of work opportunities for young women in the area; and subsequent bias in many families against having daughters.

The rationale for HCCP's innovation lies in its integration of healthcare workforce development and social empowerment within an institution already deeply embedded in community trust - The Duncan Hospital. The programme's eligibility framework is highly targeted, using a vulnerability scoring system that prioritises applicants from marginalised backgrounds based on caste, household income, family education level, housing status, and proximity to the health facility. This guarantees that the intervention's benefits are concentrated among the most vulnerable women who face multiple, overlapping forms of marginalisation.

HCCP's novelty is also reflected in its delivery model, which combines awareness-raising campaigns; a structured application, testing and selection process; preparatory mentoring including English language training (considering that the course curriculum and training is in English language), ongoing psychosocial support, and a guaranteed job at The Duncan Hospital based on a contractual employment bond. By guaranteeing placement at The Duncan Hospital for a minimum of two years post-graduation, the programme addresses both workforce supply stability and the individual's economic security. This multi-component and integrated approach – combining targeted recruitment, education, regulation, mentoring, financial incentives, and professional support through guaranteed employment, aligns with WHO guidance and systematic reviews as more effective retention mechanisms than single interventions (18–20).

The present analysis draws on six years of implementation data (2019–2025) based on an independent, objective evaluation of HCCP's implementation at The Duncan Hospital, Bihar, offering an opportunity to assess HCCP's potential contribution to both gender equity in education and hard-to-staff health workforce strengthening.

### Essential elements of the HCCP intervention

The HCCP programme is a multi-component intervention and has evolved since its launch in August 2019. Its structure and key components are informed by both contextual constraints in East Champaran District, Raxaul, Bihar and the operational capacity of The Duncan Hospital as the original primary implementing institution in India. The essential elements include:

#### Primary implementing institution – The Duncan Hospital

Positioning and funding the HCCP - a medical and healthcare career programme - within a reputed institution that has high acceptance and credibility in the community it works with is by itself a significant element and contributes to its success. This is evidenced by its achievements discussed later in the results as overtly expressed by the parents and community members.

The Duncan Hospital, established in 1930, provides healthcare to people in Bihar and neighbouring Nepal, in its strategic location at the border of Northern Bihar and Nepal. Besides being a renowned and reputed healthcare and teaching institution, in line with the mission and vision of EHA, The Duncan Hospital also has well-developed community health and development programme (CHDP) initiatives focussing on empowerment of vulnerable and marginalised groups including: women and adolescent girls from vulnerable communities; people with disabilities, mental disorder or HIV/AIDS; community organisation and advocacy for sexual and reproductive rights; protecting vulnerable families from slavery and promoting sustainable freedom; capacity-building of the government health system; and primary healthcare through clinics in peripheral health centres and mobile clinics in 3 blocks (16, 17).

The Duncan Hospital serves as both the original implementing institution and the primary employment site for its HCCP graduate young women. The hospital's established reputation for medical quality, hygiene and ethical practice has cultivated deep community trust, making it uniquely positioned to recruit young women from conservative, low-income households that may otherwise restrict daughters from travelling, living away from home for study and/or pursue employment. The local language (Bhojpuri), community ties and institutional credibility are instrumental in HCCP retention outcomes.

#### Selection criteria

All young women supported by HCCP are local to their future job, and the eligibility and selection criteria are based on their vulnerable origins. EMMS's definition of “vulnerable” includes: low familial income and lack of or low educational attainment of parents, along with the criterion of the family home being near the hard-to-staff health facility. Specifically for the India HCCP programme, the selection criteria include vulnerability factors in a scoring system based on caste, having at least one sister, low income, a preference for daily wage labourers, assessment of the land the family owns, and whether they own or rent their home.

#### Healthcare accredited educational courses offered

Various courses are offered to the young women under the HCCP programme:

Auxiliary Nurse Midwife (ANM): 2-year course
General Nurse Midwife (GNM): 3-year course
Diploma in Medical Laboratory Technology (DMLT): 2-year course
Diploma in Pharmacy (DPharma): 2-year diploma course

### HCCP delivery mechanism and process

The HCCP programme comprises interconnected stages, each of which has been developed to optimise selection, preparation or retention of the vulnerable young women:

#### Awareness-raising and targeted advocacy

HCCP Awareness activities are carried out in the local communities throughout the year in collaboration with the CHDP staff of The Duncan Hospital. These activities target parents, community leaders and potential applicants, focusing on the value of educating girls, professional healthcare careers, and the availability of HCCP support. Channels include community meetings, notice boards at The Duncan Hospital, and word-of-mouth dissemination via alumni and current beneficiaries.

#### Application and selection process

The annual application cycle (February-March) involves the distribution of application forms, which includes relevant sections for collection of detailed socio-economic data to assess eligibility. A vulnerability scoring system and screening procedure has been established that prioritises applications of young women from the most marginalised and vulnerable households based on the selection criteria of vulnerable women described above and includes the requirement to produce evidence that these young women have already achieved sufficient school results to satisfy this aspect of entrance to colleges. Eligible candidates undertake a written examination assessing English proficiency and general knowledge relevant to societal and environmental issues, which are based on the competency requirements of the accredited healthcare professional courses offered including English language competence. The academic results-based eligibility criteria for admission differ for various courses offered and respective colleges. Hence, the application form and selection process have been revised over the years to adapt to the requirements of college entrance exams and to ensure recruitment of the most vulnerable, needy and deserving young women.

#### Mentorship and preparatory training

Successful applicants receive structured mentorship to prepare for competitive college entrance examinations and interviews. This mentorship addresses linguistic competence, confidence building and interview skills that have been identified as critical determinants of admission success.

#### Financial support

HCCP offers partial financial assistance for the college tuition fees to the selected candidates, with the expectation that families contribute the remaining amount through personal resources and/or loans (e.g. Bihar Student Credit Card scheme), or other means. This cost-sharing model is intended to foster family ownership of the educational investment while enabling the programme to support a greater number of young women from the area.

#### Ongoing follow-up and psychosocial support

Throughout the studies, regular follow-up from HCCP staff is ensured via home visits, phone calls and liaison with college faculty. This monitoring serves dual purposes of academic oversight and emotional support for young women, many of whom are living away from home in college hostels or other residential facilities for the first time to pursue their education.

#### Guaranteed jobs and employment bond

Upon successful completion of their healthcare course and training, graduates enter into a two-year employment contract with The Duncan Hospital. This guarantees that the hospital benefits from a reliable supply of committed, locally rooted, culturally competent staff while providing graduates with stable employment and income in their local geographic area, professional development opportunities, and experience in a reputable institution.

### The setting: geographic context and reach

The HCCP programme is implemented in the East Champaran District of Bihar, India - a state characterised by some of the country's lowest human development indicators, persistent gender inequities, and under-resourced health infrastructure. East Champaran's estimated population exceeds four million, distributed across 27 administrative blocks, each with approximately 150,000 residents. Despite this size, access to quality healthcare remains constrained by shortages of trained personnel, especially in rural, poorer and border areas.

HCCP currently focuses on three proximal blocks but aspires to expand across the district's 27 blocks. While HCCP operates district-wide in principle, geographic reach has thus far been concentrated in three blocks nearest to The Duncan Hospital. Communities located at greater distances, especially those without CHDP presence, remain underrepresented among applicants, underscoring a challenge in equitable outreach and access to the programme.

### Conceptual framework

HCCP's design is consistent with workforce development frameworks that link educational access, local recruitment, and retention incentives to rural health system strengthening. The programme's conceptual model integrates:

Input: Recruitment of vulnerable local women; targeted financial and academic support;
Process: Mentorship, formal college education and training, and psychosocial follow-up;
Output: Qualified healthcare graduates;
Outcome: Retention in local healthcare facility and improved community health service coverage;
Impact: Enhanced gender equity and empowerment, reduced economic vulnerability, strengthened local health systems, and enhanced value of education.

Given the centrality of replication potential to EMMS's strategic vision, process-related dimensions such as scalability, replicability and sustainability are as critical to assess as end outcomes. Hence, the programme's evaluation to date has focused on both process and outcome indicators. Process indicators include number of applicants, selection ratio, mentoring participation rates, geographic coverage, and adaptations in delivery based on local context. Outcome indicators include number of graduates, retention in service post-bond, and qualitative measures of empowerment, delayed marriage, and family economic upliftment.

## Results: Key findings

Key findings, based on the thematic analysis of data, are presented below.

### Programme reach and coverage

From its inception in August 2019 to December 2025, HCCP obtained a cumulative 284 applications. Annual application volume rose from 22 in 2019 to 64 in 2025 - almost a threefold increase - reflecting increased outreach, heightened community awareness, increase in demand, and perceived value of the programme and professional education for young women. Of these applicants, 96 young women passed the initial HCCP selection test and obtained mentoring for college entrance examinations; and 54 out of 96 passed the college entrance exams to pursue various accredited professional healthcare courses. The first cohort of 24 graduate young women started employment at The Duncan Hospital in the year 2022, working in their respective fields of ANM and Lab Technician with DMLT, while others are still pursuing their courses in respective colleges (Details in Table 1).

**Table 1:** Young women part of HCCP programme so far.

Year	Total applications to HCCP	Passed HCCP screening test and received mentoring for college entrance exams	Women in various colleges^a^ pursued/ pursuing selected accredited healthcare course	Graduates working at The Duncan Hospital
2019	22	15	9 (8 ANM + 1 DMLT)	N/A
2020	35	14	8 (6 ANM + 2 DMLT)	N/A
2021	No activities due to COVID			
2022	42	13	5 (4 ANM + 1 GNM)	9 (8 ANM + 1 DMLT)
2023	59	12	7 (3 ANM + 4 GNM)	8 (6 ANM + 2 DMLT)
2024	62	22	10 (4 ANM + 4 GNM + 2 BSc Nursing)	3 (ANM interns)
2025	64	20	15 (8 GNM + 5 ANMs + 1 DPharma + 1 BSc Nursing)	3 ANMs (Interns)1 GNM
Total	284	96	54	24

a Names of the collaborating colleges/institutions have not been included to maintain confidentiality and for safeguarding reasons.

### Programme development, expansion and adaptations

The HCCP programme has expanded since its launch, with respect to the number of courses being offered as well as an increase in collaborations with various colleges and institutions, which highlights an increase in demand, value and success of the HCCP programme as well as rising awareness and value of higher education for women.

“*In the beginning, it was difficult to get girls (young women) for HCCP, however gradually girls have started reaching out themselves*”, expressed a member of the staff at The Duncan Hospital.

Expansion of courses and number of institutions and colleges has taken place since the inception of the HCCP in 2019, responding to the demand for higher qualifications and alignment with evolving hospital staffing and workforce needs. In the first and second year (2019 and 2020 respectively) of the HCCP programme, women only undertook ANM and DMLT courses, but over time GNM (in the years 2022–2025), DPharma (in response to the demand for Pharmacists by The Duncan Hospital) and BSc Nursing (2024–2025) are also offered and pursued. In order to expand the number of courses and meet increasing demand, the number of participating colleges and training institutions increased from two to multiple colleges and sites, both within and outside Bihar including other states of India such as Karnataka and Uttar Pradesh, indicating an adaptive capacity to leverage diverse educational partnerships. This also reflects shifts in community perceptions and greater mobility of women whereby the parents are willing to send their daughters to distant locations from their family homes to ensure professional higher education. As one of the HCCP staff explained:

“As the demand and intake increased, we felt a strong need to partner with more colleges and offer diverse courses, including outside Duncan area and Bihar. This included demand by The Duncan hospital for staff in diverse health sector as well as demand by the young women and community.”

Similarly several programme adaptations have emerged over the six-year period, reflecting responsiveness to operational challenges. For example, between 2019 and 2023, mentoring involved daily in-person English language and college entrance exam preparation classes over a three-month period. Responding to the geographic distance and access challenges for young women, mentorship delivery adapted to hybrid approaches, recommending local coaching centres, integrating Duolingo app for English language training and practice, and conducting online group mentoring sessions in order to help sustain participation. Graduate young women and those currently pursuing courses consistently cited the mentoring phase as a pivotal factor in admission success and confidence-building. Further, institutional integration has been strengthened. EMMS is building a new College of Nursing at The Duncan Hospital capable of teaching BSc, to replace the hospital's old School of Nursing where only GNM was being taught.

### High educational attainment with minimal attrition

The mindset of young women absorbed within HCCP, their families and communities towards education and employment of females appears to be changing. For example, before implementation of the HCCP, the majority of the families would get their girls married after school (class XII) while a few of them allowed their daughters to study up to graduation at the most, but the thought of higher education and/or employment for girls did not persist. However, as an impact of the HCCP, the value of education is being recognised and accepted, and many young women express a desire to pursue nursing education and are receiving family support for the same learning from the positive experiences and life style of the HCCP-supported young women.

“Initially people in my neighbourhood used to say to my parents why are you getting your daughter educated, what will she do after studying, after all she has to get married but now looking at me and other HCCP-supported young women, they regret marrying their daughters early and not educating their girls.” - HCCP graduate young woman

It is further reported that parents with higher economic status are spending money and investing in the education of their daughters.

“Now, looking at my daughter who is successfully employed as a Nurse at The Duncan Hospital, many parents in the community are funding education of their daughters and want them to work as a Nurse.” – Parent of a HCCP graduate young woman

Similarly, another parent shared that, *“Getting inspired by my daughter, her private coach/tutor has sent both his children to study GNM at his own expense.”* – Parent

Inspired by the current HCCP Nursing graduate young women, a greater number of girls in the community are now choosing science subjects in schools, as it is one of the eligibility criteria for BSc Nursing. Even boys are motivated and inspired to pursue education including medical, healthcare and/or nursing education courses. Further, aspirations for higher education are growing for those who have received HCCP support. A number of parents expressed their desire for their daughters to continue higher education after the HCCP graduate course, and some young women and families expressed keenness to explore ways to fund that.

“We want our children to study further, from ANM to GNM to BSc, it should not stop here (referring to HCCP support received for one course)”- Parent

All HCCP-supported young women and their families expressed their desire for the HCCP programme to continue and expand affirming that, “*now awareness has been created, and people want to send their daughters for education, so the HCCP should continue*.” – Parent

It is being widely recognised that educating girls and women will lead to generational change as well as to the development of the communities and nation as a whole – not only the single woman and her family, as expressed by a parent, “*The change will trickle down from one generation to the other by educating the girl child*.”

Attrition in the HCCP-supported education and training has been minimal. Only one young woman pursuing an ANM course discontinued her course due to health concerns, with plans to rejoin in the following year. Delays in institutional registration affected two GNM students in 2024, but they expressed intent to reapply.

### Employment outcomes, workplace integration and career progression

The HCCP nurses are growing as strong professionals and are being accepted and considered as “*long-term staff with good skills, which is an asset for the hospital*” (Staff), which is a sentiment echoed by many staff members interviewed for the evaluation.

All graduates to date have entered the guaranteed two-year employment bond at The Duncan Hospital. Feedback received from the hospital administrators and supervisors during the evaluation interviews emphasises the benefits of this arrangement including predictable staffing, reduced turnover, and value of these HCCP-supported and recruited young women's fluency in the local Bhojpuri language. HCCP graduate young women are considered to be committed, disciplined and adaptable. As reported by the staff, their linguistic competence in Bhojpuri facilitates rapport with patients, complementing the skills of non-local medical staff. Besides, all HCCP graduate young women receive targeted on-the-job training to address gaps, if any, e.g., technical documentation and communication needs. An inclusive workplace culture fosters equal treatment alongside other staff.

“The girls do very well. They are hardworking, disciplined, understanding; and to date we have had no problem.” – College Principal

Early evidence suggests strong post-bond retention, with the majority of the graduate young women continuing employment voluntarily beyond the contractual period. Career progression is also reflected for three young women who moved on from The Duncan Hospital, as two graduates left work to pursue further higher studies and another is now working with the Bihar government.

### Economic outcomes and household impact

Qualitative interviews underscore the transformative economic effects of HCCP participation. All families reinforced their financial struggles and economic hardships that impeded their ability to educate their daughters even if they wished to. Within this context, HCCP support provided relief and a path for success and growth.

My daughter would not have been able to study if not for HCCP support as her father is paralysed and I only earn Rs. 3000 per month (approx. £25) - Mother of a HCCP-supported YW, which is a sentiment expressed by many family members.

“None of the parents had the financial capacity to get their daughters educated if not for the HCCP support” – Parents

Similarly, a few parents attributed their focus on getting their daughters married early to ignorance, lack of knowledge, and/or guidance. HCCP not only helped households by raising awareness, it also created tangible opportunities for young women, as expressed by a parent, “*Earlier we were ignorant and the only way forward for daughters seemed to be to get them married, but HCCP showed us a path*.”.

Economic status and stability of the families is reported to be improving as HCCP-supported young women now working at The Duncan Hospital contribute significantly to household income, support siblings' education, and in some cases serve as the primary earners.

“Before the age of 18, I am independent and contributing financially to my family. I feel proud of myself. I will turn 19 in August. I support my siblings too. Now my sisters have gone to study.” - HCCP graduate young woman working at The Duncan Hospital

Parents reported relief from financial pressure, reduced dowry obligations, reduced pressures in marriage negotiations, and increased household stability as an outcome of their daughter's employment at The Duncan Hospital due to the HCCP support. Case examples include: HCCP graduate young women supporting families after a parent's or sibling's incapacitating illness or loss of a parent; a few of them are supporting a sibling's education; and one young woman is funding her sister's nursing education - initiating an intra-family multiplier effect.

“After completing my education and working, now I am the right hand of my father and he appreciates me like his son, whereas earlier he did not want me to study or work, but one of the HCCP staff convinced him. Now he feels very happy and proud of me” - HCCP graduate young woman working at The Duncan Hospital

### Personal and social empowerment

“Because of HCCP, girls have got wings to fly.” - Parent of HCCP-supported young woman

Young women recipients and their families described notable shifts in self-perception, confidence and women's agency.

“Doing the HCCP-supported ANM course is like the biggest gift in my life. God picked me up from a drain and took me up, and HCCP showed me a path; no one can pull me down now, it is only going to be an upward journey” – HCCP graduate young woman//current student

HCCP-supported young women graduates reported improved communication skills, capacity to make independent decisions, and greater respect within both family and community contexts. Further, parents and community members reported observing behavioural changes among the HCCP-supported women, such as improved self-presentation, assertiveness and the ability to articulate personal goals as well as willingness and skills in offering medical or nursing care to family and community members when required. HCCP graduates are increasingly regarded as role models, inspiring peers and younger children to pursue science education and professional careers, reflecting positive transformations in intergenerational educational aspirations.

Now people listen to us, and we guide them about letting their children study and pursue the field they like. People seek our advice now. – HCCP-supported young woman/current student

### Community and socio-cultural outcomes

The programme has contributed to shifts in community perceptions, attitudes and practices:

#### Delayed marriage and childbearing

Earlier, prior to the HCCP programme, girls in the community were married at an early age of 14–16 (or earlier) against their will, and without their consent and/or opinion. However, due to the HCCP intervention, many young women remain unmarried throughout their education and training and initial employment period, and families are able to resist the community pressure for marriage. This is leading to a transformation within the community whereby a greater number of parents and women are aspiring to and applying for HCCP support for professional education and employment prospects, rather than considering marriage as the only option.

“As soon as girls grow up, the society is more concerned about our daughter than us as parents, and there is a social pressure to get them married. But this HCCP programme is enabling us to take a stand against it and letting our daughters study and work” – Parent

Since girls are considered a burden, marriage is generally seen as a way of “*getting rid of this burden by getting her married, but this is changing now due to HCCP*” – a sentiment expressed by a parent of a HCCP graduate young woman, and echoed by many family and community members.

Further, often women in the community conceived a child soon after getting married, often unplanned, and delay in childbearing is also reported among HCCP-supported married women with changing mindsets of their families, including their husbands and parents-in-law.

“Now these young women are thinking of career progression and don't want to start a family or have a child soon even if they get married after starting work at The Duncan Hospital.” Staff

#### Reduced dowry demands and shifts in relationship patterns

Reduction in dowry demands and expectations are also reported. Families of HCCP-supported graduate young women receive marriage proposals with lower or no dowry expectations, often from better-educated suitors and prospects. Many parents during FGDs expressed a relief with this stating:

“Before the HCCP-supported education and job for girls, boys were demanding dowry as high as 12,00,000 (approx. £10,000). Now we see that no or very less dowry demands are being made; the boys and their families are seeking girls who are educated and/or working as nurses.”

Positive shifts in relationship patterns are also being noted, with husbands of HCCP-supported women being more collaborative and engaged in household chores and attempting to move closer to their wife's place of work (The Duncan Hospital) due to their greater job stability, reflecting acceptance and respect of women as earning members of the household.

#### Shifts in gender norms

Change in the overall gender norms and acceptance of a girl child are reported as an impact of the HCCP – with noted shifts in considering a girl child as a “*burden*” previously to accepting and even welcoming them as “*productive*” beings – reflecting changing social status of females in the community.

“People are beginning to look at girls as productive, rather than a burden, which is changing the social status of the girls” - Medical Staff, Duncan Hospital.

Increasing acceptance of daughters' mobility, education, employment and financial independence is also reported, with growing trust in their autonomy, and reduced stigma towards working women and their families.

“People tell us you are living on daughter's income, but we don't pay attention any more, we keep our ears closed to such comments now”. – Parent

As shared by respondents, many HCCP young women are the first ones in their families and/or villages to pursue higher/professional education and work as a nurse, which is also potentially contributing to changing gender norms. This is also leading to increased professional aspirations for working women, with families wanting their daughters or even daughters-in-law to continue working after marriage, which has been a stigma for many women impeding their economic participation and employability. Many parents have started affirming that their daughter will continue to work after marriage, and they are refusing such proposals where the boy's family does not accept it.

“I told the boy’s family clearly that I and my daughter have put a lot of effort and hard work to ensure her education and nursing job, and she will not give it up for anything, even after her marriage.” – Parent (Mother)

### Challenges identified

Despite positive outcomes, several implementation challenges with HCCP were reported:

Limited outreach beyond immediate hospital-adjacent communities, as a staff member reported, “*Many blocks have not been touched at all for girls in other areas*.”
Partial fee coverage for courses undertaken, although the most financially constrained families will have full fees paid as an outcome of the evaluation findings;
Mentoring limitations related to distance, digital access, and variable quality of local coaching;
Course portfolio narrowness, with heavy focus on nursing and limited awareness of alternative career paths;
Follow-up gaps for unsuccessful applicants, many of whom revert to early marriage or unrelated employment due to lack of economic, mentorship, and employment support as provided by the HCCP programme.

## Discussion

The HCCP programme illustrates that a targeted, locally anchored intervention can simultaneously address rural, remote or resource-poor healthcare workforce shortages and structural gender inequities in education and employment. Critically influential features that distinguish HCCP from conventional scholarship schemes, are discussed. A cohesion of these elements constitutes HCCP's operational framework ensuring cultural alignment and community trust, which is central to understanding its reported outcomes. Its approach is consistent with the World Health Organisation guidelines for workforce recruitment and retention including the ways in which students are selected and educated, as well as creating better working and living conditions (18, 19), and is supported by India-specific evidence on mentoring and retention (12, 21–23). It also corresponds with India's National Health Policy (NHP) 2017 that recommended advancing the education system and the development of a cadre of healthcare providers (6). Investing in health workforce education and development is also emphasised by the WHO as a pathway to creating employment opportunities for women and achieving the sustainable development goals, particularly in LMICs (2, 3).

HCCP's integration within a respected healthcare institution - The Duncan Hospital - is central to the young women's participation as it has helped in overcoming parental and community reluctance to allow daughters to study away from home or pursue work before or even after marriage. This is consistent with the literature emphasising institutional credibility as a determinant of participation in gender-sensitive interventions in conservative settings - a factor recognised in Indian qualitative studies of rural workforce recruitment (24).

Targeted selection and vulnerability scoring ensures resources are allocated to women facing compounded disadvantages, increasing the likelihood of transformative personal and household impact. HCCP's approach aligns with the literature that emphasises the need to tackle gender inequalities intersectionally, such as engaging vulnerable women to address issues of education including mentoring and skill-building, healthcare and workforce limitations, and poor economic independence (4).

Another distinguishing feature is the integrated mentorship model focusing on both academic readiness and psychosocial resilience. In the Indian context, nurse mentoring initiatives such as AMANAT and other in-service mentoring programmes have provided empirical precedent demonstrating the value of mentorship in improving clinical skills, readiness and trainee confidence (12, 21, 22), supporting HCCP's mentorship emphasis.

Employment guarantee through a service bond provides financial security and professional integration, while meeting local staffing needs. HCCP's success in retaining graduates within The Duncan Hospital is consistent with broader evidence that local origin, community trust, and locally bonded employment contracts improve staff retention in underserved areas. While bonding secures short-term staffing, literature on compulsory service in India and globally indicates that bonds are most effective when combined with supportive working conditions, incentives and professional development opportunities (25–27). While professional integration through support and on-the-job-training is ensured through follow-up within HCCP, support for further professional development opportunities could be considered and strengthened.

The distinguishing features of HCCP discussed above work synergistically to create a self-reinforcing cycle of empowerment and workforce stability. The observed socio-cultural outcomes, such as delayed marriage, reduced dowry expectations, and changing attitudes towards daughters' birth, marriage, education and autonomy, reflect the intersectional benefits of educational and economic empowerment. These impacts extend beyond the individual HCCP-supported young women, influencing parents, siblings, peers, and wider community norms, suggesting that HCCP operates as both a workforce intervention and a gender-transformative social programme. Aligned with Kabeer's resources–agency–achievements framework (28, 29), the HCCP programme operates as a gender-empowerment pathway embedded within health workforce strengthening. By expanding marginalised women's access to educational and financial resources, enhancing their agency through mentoring and institutional support, and translating these into accredited qualifications and secure employment, HCCP illustrates how a health workforce initiative can enhance both gender empowerment and health system-level gains (28, 29). Importantly, as evident from the findings, the HCCP also creates conditions for longer-term structural and intergenerational effects- a dimension implicit in Kabeer's framework, as women's visibility in skilled healthcare roles such as nursing, is challenging prevailing gender norms, influencing community perceptions of women's work and mobility, and raising educational aspirations for younger girls.

The replicability potential of HCCP is being demonstrated through its implementation by EMMS in other EHA sites - Jiwan Jyoti Hospital and Madhipura Hospital. As of 2025, 8 candidates at Jiwan Jyoti Hospital have already been enrolled - 2 ANM and 6 GNM - and 4 have been enrolled for GNM at the Madhipura Hospital HCCP programme. This demonstrates HCCP as an evidence-based model that has a wide scope of replication and sustainability.

HCCP's model is, in principle, replicable in other hard-to-staff contexts, provided that adaptations account for local sociocultural norms, credibility of implementing institutions, and availability of accredited colleges. The cost-sharing model for tuition enables broader reach but may limit inclusion of the most financially deprived households, suggesting that a hybrid funding mechanism (partial subsidies for most, full subsidies for the poorest) could improve equity without reducing coverage. Replication efforts could also address further diversification into allied health professions (e.g., laboratory work, pharmacy, physiotherapy, radiology, community health work), which could broaden education and employment opportunities, as will other needs of a hospital, such as IT and accountancy skills.

## Key recommendations

### Lessons learnt and practical considerations

For policymakers, NGOs and healthcare institutions, the following evidence-based recommendations emerge to scale or replicate HCCP in other settings:

Although the specific socio-cultural context of Raxaul and The Duncan Hospital's reputation are unique, many elements of the HCCP model are transferable to other healthcare settings in India and comparable LMIC contexts. Yet, successful replication would require adaptation to the country and geographic context, local institutional credibility, community norms regarding gender and female mobility, and the availability of accredited colleges and training partners for various healthcare courses.
Strengthen outreach beyond immediate institutional catchment areas (for e.g., The Duncan Hospital in this case) by partnering with schools, women's groups and local governance bodies in the more remote and underserved regions to ensure outreach to the most economically and socially vulnerable females and communities;
Considering limitations of distance and mobility while acknowledging the critical need for mentoring during the training phase, enhance mentorship accessibility through a mixed delivery model as may be required by combining centralised residential training, localised in-person sessions and structured online support, with investment in digital infrastructure;
Expanding the scope of healthcare workforce development and education beyond nursing by diversifying course offerings to include shorter-duration certifications and non-nursing health professions, which has emerged as a critical need within the HCCP programme as well. This will help engage a wider network of young women such as those without science background in schools or those who are unable to dedicate 3–4 years to gain a healthcare qualification such as BSc.
Develop structured follow-up for unsuccessful applicants (who do not clear the college entrance exam or interviews) or ineligible applicants (who do not completely fulfil the vulnerability score criteria), offering guidance into alternative education or vocational pathways to reduce vulnerability and to ensure social and economic empowerment.

### Future studies

Despite growing attention to health workforce shortages, evidence remains limited on how gender norms and institutional biases shape recruitment, retention, and career progression across health professions, and often overlook intersectional dynamics and longitudinal career pathways. Future research needs to prioritise gender-responsive workforce analytics, evaluation of policy interventions, and the role of leadership, working conditions, and governance structures in sustaining an equitable health workforce. Moreover, in order to facilitate cross-country learning on primary healthcare-oriented workforce models (2), primary research studies and systematic and realistic reviews, focussing on consolidating such models including critically monitoring and evaluating progress (19, 30) within different socio-cultural and economic contexts would be beneficial for researchers, policy makers, and practitioners.

## Conceptual and methodological limitations

While the programme evaluation presents compelling evidence of impact, certain limitations should be acknowledged:

Most beneficiaries are from communities near The Duncan Hospital, which may bias results towards higher retention due to proximity and social ties;
Data on young women graduates' career trajectories beyond the two-year bond period remain limited due to the short implementation time period, with first cohort of graduates placed in the year 2022, constraining long-term impact assessment;
The nursing-centric focus of the HCCP support limits exploration of whether similar support models could be equally effective for other health and non-health professions and workforce required in a hospital;
Incomplete counterfactual data and lack of support to unsuccessful applicants, reflecting that while anecdotal cases of early marriage among unsuccessful applicants highlight HCCP's protective role, systematic comparison is lacking;
Overall, it is a small scale study of the HCCP model's implementation in one single community, which reflects limitations of transferability, contextual specificity, and generalisability.

## Conclusion

The HCCP programme, as implemented by EMMS International in partnership with The Duncan Hospital, presents a promising, evidence-informed, integrated and context-sensitive intervention that addresses both the shortage of healthcare personnel in underserved, hard-to-staff areas and entrenched gender inequities in education and employment. Over a six-year period (2019–2025), the programme has demonstrated measurable success in enabling local, vulnerable young women to access accredited healthcare qualifications and training, and enter stable employment within their own communities. Of particular note, the programme's impact extends beyond immediate beneficiaries, influencing social norms around education of the girl child, marriage, childbirth, and professional aspirations, contributing to a gradual shift in community attitudes towards gender and work and even the desirability of having a girl child.

HCCP's dual focus on service delivery and social transformation makes it relevant not only to healthcare workforce planners but also to policymakers and practitioners engaged in gender and development programming. Sustained commitment to its core principles, alongside strategic enhancements, could enable HCCP to serve as a replicable model for health system strengthening in India and beyond.

## Data Availability

The data analysed in this study is subject to the following licenses/restrictions: N/A. Requests to access these datasets should be directed to corresponding author Dr Javita Narang (javita_narang@yahoo.co.uk) or Dr Cathy Ratcliff, CEO, EMMS International (cathy@ratcliff.scot).
